# Draft Genome Sequences of Four Commensal Strains of *Staphylococcus* and *Pseudomonas* Isolated from Healthy Human Skin

**DOI:** 10.1128/MRA.01032-20

**Published:** 2021-01-07

**Authors:** Xavier Janvier, Amine M. Boukerb, Marc G. J. Feuilloley, Anne Groboillot

**Affiliations:** a Laboratory of Microbiology, Signals and Microenvironment LMSM EA 4312, University of Rouen-Normandy, Normandy University, Evreux, France; University of Rochester School of Medicine and Dentistry

## Abstract

*Staphylococcus* spp. and *Pseudomonas* spp. are widely distributed bacteria in the environment and are found in association with animals and humans. Here, we present the draft genome sequence data of the healthy human skin commensal strains Staphylococcus aureus MFP03, Staphylococcus epidermidis MFP04, Staphylococcus capitis MFP08, and Pseudomonas fluorescens MFP05.

## ANNOUNCEMENT

*Staphylococcus* and *Pseudomonas* are among the most abundant genera of *Firmicutes* and *Proteobacteria*, respectively, which are major phyla of the skin microbiota (1–3). Although Staphylococcus aureus, Staphylococcus epidermidis, and Staphylococcus capitis are species that reside abundantly on the skin (4), they are described mainly as pathogens. Therefore, it seems relevant to sequence skin commensal strains of these species. Moreover, no sequences of Pseudomonas fluorescens cutaneous commensal isolates had been reported yet.

In healthy individuals, skin microbiota bacteria are harmless to the host and play a central role in skin homeostasis (5); therefore, they should possess few virulence factors. Nevertheless, exoenzymes, often considered virulence factors in pathogens (6), are also secreted by commensals contributing to host innate defense mechanisms (7). Here, we report the draft genome sequences of four skin commensal bacterial strains previously isolated from healthy volunteers (8), i.e., Staphylococcus aureus MFP03, Staphylococcus epidermidis MFP04, Staphylococcus capitis MFP08, and Pseudomonas fluorescens MFP05. A particular focus was given to virulence and exoenzyme genes.

Cryo-frozen isolates were grown 24 h in LB medium at 180 rpm and 37°C for *Staphylococcus* strains or at 28°C for the Pseudomonas fluorescens MFP05 strain. Genomic DNA was extracted using the GeneJET genomic DNA purification kit (Thermo Fisher Scientific, USA), following the manufacturer’s instructions, directly on the Pseudomonas fluorescens pellet or after a 60-min treatment of *Staphylococcus* strain pellets with lysis solution (400 µg/ml lysostaphin, 20 mg/ml lysozyme, 20 mM Tris-HCl [pH 8.0], 2 mM EDTA, and 1.2% Triton X-100). The quality and concentration of DNA were determined on a Nanodrop spectrophotometer and a Qubit 4.0 fluorometer (Thermo Fisher Scientific). Libraries were prepared using the Illumina Nextera XT or the Nextera Flex DNA library prep kits (Table 1) and sequenced on the MiSeq platform (Illumina) according to the manufacturer’s protocol, with the MiSeq reagent cartridge V3 (600 cycles, 250-bp or 300-bp dual-index paired-end reads).

**TABLE 1:** Sequencing metrics and genomic data

Parameter	Data for:			
	S. aureus MFP03	S. epidermidis MFP04	*S. capitis* MFP08	P. fluorescens MFP05
Site of isolation	Human, cheekbone	Human, cheekbone	Human, scapula	Human, scapula
DNA library prep kit (bp)	Nextera XT (2 × 250)	Nextera Flex (2 × 300)	Nextera XT (2 × 250)	Nextera XT (2 × 250)
Sequencing metrics				
No. of reads	899,406	854,508	874,656	1,812,096
Mean coverage (×)	79.37	95.08	86.39	59.6
Accession no.				
GenBank	JACFUB000000000	JACFUA000000000	JACFTY000000000	JACFTZ000000000
SRA	SRR12339019	SRR12339018	SRR12339016	SRR12339017
Genomic data				
Genome size (bp)	2,694,498	2,477,229	2,471,586	6,610,034
G+C content (%)	32.77	31.94	32.71	59.83
No. of contigs	25	48	50	100
*N*_50_ value (bp)	607,734	256,159	249,048	250,027
No. of CDS^*a*^	2,484	2,309	2,340	6,072
No. of tRNAs	59	49	59	56
No. of rRNAs	4	4	5	2
CheckM				
Completeness (%)	98.82	99.61	99.81^*b*^	99.53
Contamination (%)	0.48	0.00	0.10^*b*^	1.46
Strain heterogeneity (%)	0.00	0.00	0.00^*b*^	6.25
Virulence factors(s)^*c*^	*adsA*, *aur*, *cap8A* to *cap8P*, *chp*, *clfAB*, *ebp*, *T7SS*, *fnbAB*, *hly/hla*, *hlb*, *hld*, *hlgABC*, *icaA* to *icaD*, *sspA*, *sspBC*, *isd*, *hysA*, *sbi*, *geh*, *coa*, *spa*, *map*, *scn*, *sak*, *vWbp*	None	None	None
Antibiotic resistance(s)^*d*^	*blaZ*, *lmrS*, *mepA*, *mepR*, *tet*(38), *norA*, *dha1*, *aph*	*blaZ*, *dfrC*, *fosB*, *norA*	*norA*	*mexF*
MLST profile^*e*^	45	65	NA	111^*f*^
Exoenzymatic activities^*g*^				
Lipase/esterase	+	+	+	+
Urease	−	−	−	+
Sialidase	−	−	−	−
Hyaluronidase	+	−	−	−
Sphingomyelinase	−	−	−	−
Ceramidase	−	−	−	−
Protease	+	+	+	+

a CDS, coding DNA sequences.

b CheckM analysis achieved at the genus level due to the absence of the *S. capitis* rank.

c *adsA*, adenosine synthase A; *aur*, zinc metalloproteinase aureolysin; *cap8A* to *cap8P*, capsular polysaccharide synthesis enzymes A to P; *chp*, chemotaxis inhibitory protein; *clfAB*, clumping factor A fibrinogen-binding protein, clumping factor B adhesin; *ebp*, cell surface elastin binding protein; *T7SS*, type VII secretion system; *fnbAB*, fibronectin-binding proteins A and B; *hly/hla*, alpha-hemolysin; *hlb* and *hld*, beta and delta-hemolysins; *hlgABC*, gamma-hemolysin components A to C; *icaA* to *icaD*, intercellular adhesion proteins A to D; *sspA*, V8 protease; *sspBC*, staphopain B and C; *isd*, iron-regulated surface determinant; *hysA*, hyaluronate lyase; *sbi*, staphylococcal binder of immunoglobulin; *geh*, glycerol ester hydrolase; *coa*, staphylocoagulase; *spa*, protein A; *map*, extracellular proteins Map; *scn*, staphylococcal complement inhibitor; *sak*, staphylokinase; *vWbp*, von Willebrand factor-binding protein; none, absence of virulence genes.

d *blaZ*, beta-lactamase; *dfrC*, trimethoprim; *fosB*, fosfomycin; *lmrS,* aminoglycoside diaminopyrimidine macrolide oxazolidinone phenicol; *mepA mepR*, glycylcycline tetracycline; *mexF*, diaminopyrimidine fluoroquinolone phenicol; *norA*, fluoroquinolone; *dha1*, cephalosporinase; *aph*, aminoglycoside phosphotransferase; *tet*(38), tetracycline.

e Based on pubmlst schemes (https://pubmlst.org/). NA, no scheme available.

f New ST described in this work with *gyrB*(22) and the new alleles *glnS*(98), *ileS*(93), *nuoD*(98), *recA*(94), *rpoB*(87), *rpoD*(84).

g The symbols + or − indicate presence or absence, respectively, of coding genes involved in the corresponding enzymatic activity.

Default parameters were applied for all software unless otherwise specified. FastQC v.0.11.9 (9) was utilized to check read quality, and Trimmomatic v.0.39 (10) was used to quality trim the generated reads. Genome assembly was achieved *de novo* with Unicycler v.0.4.8 (11), and CheckM v.1.1.3 (12) was used to assess contamination. QUAST v.5.0.2 (13) was used to check the consistency of the obtained assemblies (i.e., genome size, number of contigs, *N*_50_ value, and G+C content). Annotation was carried out using the NCBI Prokaryotic Genome Annotation Pipeline (PGAP) (14). Sequence type (ST) identification was performed using the program MLST v.2.19.0 (15). The contig-based search method abricate v.0.8.13 was used to identify virulence factors and antibiotic resistance genes (16). Custom abricate databases were created for the following enzymes, based on downloaded NCBI reference sequences from *Pseudomonas* and *Staphylococcus* keywords: lipase, esterase, urease, sialidase, hyaluronidase, sphingomyelinase, ceramidase, and protease. The obtained metrics and results are presented in Table 1.

These draft annotated genome sequences of human skin isolates will improve the understanding of genetic diversity and enzymatic activities as well as the mechanisms involved in microbiota-human skin interactions.

### Data availability.

MiSeq sequencing reads and draft genome assemblies and annotations have been deposited in the Sequence Read Archive (SRA) and GenBank, respectively, under the accession numbers listed in Table 1. The abricate custom databases used in this study are available from the corresponding authors upon request.
